# Prevalence and management of proteinuria associated with vascular endothelial growth factor receptor‐targeted tyrosine kinase inhibitor treatment in advanced renal cell carcinoma, hepatocellular carcinoma, and thyroid cancer

**DOI:** 10.1111/iju.15409

**Published:** 2024-02-06

**Authors:** Taigo Kato, Ryuichi Mizuno, Hideaki Miyake

**Affiliations:** ^1^ Department of Urology Osaka University Graduate School of Medicine Osaka Japan; ^2^ Department of Urology Keio University School of Medicine Tokyo Japan; ^3^ Division of Urology Kobe University Graduate School of Medicine Hyogo Japan

**Keywords:** carcinoma, renal cell, proteinuria, receptors, tyrosine protein kinase inhibitors, vascular endothelial growth factor

## Abstract

Vascular endothelial growth factor receptor‐targeted tyrosine kinase inhibitors (VEGFR‐TKIs) are often used for treatment of several types of cancer; however, they are associated with an increased risk of proteinuria, sometimes leading to treatment discontinuation. We searched PubMed and Scopus to identify clinical studies examining the incidence and risk factors for proteinuria caused by VEGFR‐TKIs in patients with renal cell carcinoma, thyroid cancer, and hepatocellular carcinoma. The global incidence of proteinuria ranged from 6% to 34% for all grades of proteinuria, and from 1% to 10% for grade ≥3 proteinuria. The incidence of proteinuria did not differ significantly by cancer type, but in all three cancer types, there was a trend toward a higher incidence of proteinuria with lenvatinib than with other VEGFR‐TKIs. In terms of risk factors, the incidence of proteinuria was significantly higher among Asians (including Japanese) compared with non‐Asian populations. Other risk factors included diabetes mellitus, hypertension, and previous nephrectomy. When grade 3/4 proteinuria occurs, patients should be treated according to the criteria for dose reduction or withdrawal specified for each drug. For grade 2 proteinuria, treatment should be continued when the benefits outweigh the risks. Referral to a nephrologist should be considered for symptoms related to decreased renal function or when proteinuria has not improved after medication withdrawal. These management practices should be implemented universally, regardless of the cancer type.

**Abbreviations & Acronyms**
CTCAE: Common Terminology Criteria for Adverse Events
eGFR: estimated glomerular filtration rate
HCC: hepatocellular carcinoma
ICI: immune checkpoint inhibitor
RAS: renin–angiotensin system
mRCC: metastatic renal cell carcinoma
RR: relative risk
TC: thyroid cancer
TKI: tyrosine kinase inhibitor
UPCR: urinary protein creatinine ratio
VEGFR: vascular endothelial growth factor receptor

## INTRODUCTION

Vascular endothelial growth factor receptor‐targeted tyrosine kinase inhibitors (VEGFR‐TKIs) are the primary treatment for a variety of cancers. Particularly in metastatic renal cell carcinoma (mRCC), the combination of immune checkpoint inhibitors (ICIs) and VEGFR‐TKIs is the standard first‐line treatment approach.^1^ However, VEGFR‐TKIs are associated with an increased risk of proteinuria,^2^ which is a direct indicator of nephrotoxicity.^3^ Such nephrotoxicity can also occur with new regimens that include TKIs.^4^ Although these nephrotoxic effects are mostly mild in severity, they sometimes lead to treatment discontinuation.^3^

Multiple pathways are thought to be associated with the development of proteinuria associated with VEGFR‐TKIs.^5^ Loss of normal regulation of VEGF‐A expression results in structural and functional changes, including impaired capillary endothelial opening, glomerular endothelial cell proliferation (endotheliosis), and loss of podocytes.^6^, ^7^, ^8^ Antagonism of VEGF also leads to dysregulation of renal repair processes in the early stages of renal injury and promotes glomerulosclerosis. Furthermore, inhibition of VEGF signaling can result in selective loss of glomerular permeability.^9^, ^10^ The occurrence of proteinuria can be a disincentive to continue with cancer treatment. Therefore, it is important to understand the risk of proteinuria in different types of cancer and to manage this risk appropriately.

Racial differences are apparent in the nephrotoxicity of VEGFR‐TKIs, with the risk being higher in Asian people.^11^ However, it is unclear to what extent the occurrence of proteinuria in Asians, especially those of Japanese ethnicity, is actually increased by VEGFR‐TKIs. It is also unclear whether there are differences among VEGFR‐TKIs and whether the type of cancer influences the likelihood of proteinuria development. The combination of VEGFR‐TKIs and ICIs is recommended as the first‐line treatment regimen for mRCC; therefore, the occurrence of proteinuria with this regimen should be better understood.

We conducted a narrative review to examine the incidence of proteinuria in clinical trials of VEGFR‐TKIs, focusing on the treatment of mRCC, in which VEGFR‐TKIs play a central role as a systemic therapy. We also included reports related to thyroid cancer (TC) and hepatocellular carcinoma (HCC). Moreover, we conducted a narrative review to examine the incidence of proteinuria based on clinical trials of VEGFR‐TKIs, including reports related to HCC and TC. We also examined the factors that increase the risk of proteinuria, as well as the approaches used to manage proteinuria in patients undergoing VEGFR‐TKI treatment.

## STUDY SELECTION

We conducted a narrative review to examine the incidence of proteinuria caused by VEGFR‐TKIs and to identify risk factors associated with proteinuria by searching PubMed and Scopus.

### Cancers and drugs included in this search

The carcinomas searched were mRCC, TC, and HCC because they are the cancers most commonly treated with VEGFR‐TKIs. The drugs searched were axitinib, pembrolizumab, avelumab, cabozantinib, nivolumab, lenvatinib, everolimus, pazopanib, and sunitinib for mRCC; lenvatinib, cabozantinib, and vandetanib for TC; and sorafenib, lenvatinib, regorafenib, and cabozantinib for HCC. These drugs are recommended in the National Comprehensive Cancer Network or European Society for Medical Oncology guidelines. Regimens combining VEGFR‐TKIs with ICIs are used in clinical practice for mRCC; therefore, these regimens were also included in the search.

### Previous reports on incidence of proteinuria included in this search

Phase 3 trials of VEGFR‐TKIs or VEGFR‐containing multikinase inhibitors and phase 2 trials on Japanese and Asian patients and their subgroup analyses were included in this narrative review of the incidence of proteinuria. In these reports, proteinuria was evaluated based on the Common Terminology Criteria for Adverse Events (CTCAE) versions 3.0–5.0. Grade 4 proteinuria, defined in CTCAE version 3.0, was reclassified as nephrotic syndrome in version 4.0 and subsequent versions.

### Previous reports on risk factors for proteinuria with VEGFR‐TKIs and time course of proteinuria and renal function associated with VEGFR‐TKIs in this search

This narrative review included phase 1–3 trials and prospective and retrospective observational studies on the risk factors for proteinuria development with VEGFR‐TKIs, as well as the transition to proteinuria and renal dysfunction with VEGFR‐TKIs. The incidence of proteinuria associated with VEGFR‐TKIs was examined to determine whether there is an effect of cancer type, whether there are differences in the incidence of proteinuria among VEGFR‐TKI drugs, and whether there are racial differences in the incidence of proteinuria, particularly among Asian populations, including Japanese people.

## FREQUENCY OF PROTEINURIA WITH VEGFR‐TKIS

### Comparison of cancer types

The global incidence of proteinuria ranged from 6% to 34% for all grades, and from 1% to 10% for grade ≥3. There was no marked difference among the three types of cancer in terms of the incidence of proteinuria with VEGFR‐TKIs (Table 1).^12^, ^13^, ^14^, ^15^, ^16^, ^17^, ^18^, ^19^, ^20^, ^21^, ^22^, ^23^, ^24^, ^25^, ^26^, ^27^, ^28^, ^29^, ^30^, ^31^, ^32^, ^33^, ^34^, ^35^, ^36^, ^37^ In a meta‐analysis (*n* = 9446) examining the incidence of proteinuria in clinical trials of five VEGFR‐TKIs (regorafenib, vandetanib, cabozantinib, lenvatinib, and axitinib) in patients with multiple carcinomas, including mRCC and HCC, the use of VEGFR‐TKIs significantly increased the relative risk (RR) of all‐grade proteinuria (RR 2.35, 95% confidence interval [CI] 1.69–3.27, *p* < 0.001) and high‐grade proteinuria (RR 3.70, 95% CI 2.09–6.54, *p* < 0.001).^2^ For each cancer type, the RR of proteinuria with VEGFR‐TKIs compared with the control arm was higher for HCC and mRCC for all‐grade proteinuria, and significantly higher for HCC and mRCC for high‐grade proteinuria. There was no statistically significant difference in the RR by cancer type, although the frequency of proteinuria among the cancer types tended to be higher for HCC and mRCC.^2^ Similar to the results of the previous meta‐analysis,^2^ no significant trend in the frequency of proteinuria was observed among the different cancer types. Furthermore, the relationship between the grade of proteinuria and treatment response remains unclear, with some research indicating a positive correlation with overall survival after TKI treatment,^38^ and other research finding no correlation with progression‐free or overall survival.^39^

**TABLE 1:** Incidence of proteinuria in the clinical trials of VEGFR‐TKIs: Summary.

Tumor type	Treatment	Proteinuria, %				Ref
		Global		Japanese (Asian)		
		Any grade	Grade ≥ 3	Any grade	Grade ≥ 3	
mRCC	Axitinib	11–23	—	12–64	4–9	12, 13, 14, 15, 16
	Axitinib + pembrolizumab	17.5	2.8	40.9	4.5	17, 18
	Axitinib + avelumab	6	1.6	18.2	0	19
	Cabozantinib	14	2	40	8.6	20, 21
	Cabozantinib + nivolumab	10.3	2.8	—	—	22
	Lenvatinib + pembrolizumab or everolimus	29.5–34.1	7.7–8.2	54.8	14.3	23, 24
	Pazopanib	18	4	31 (A)		25, 26
	Sunitinib	7.8–14	1.4–4	29 (A)–72	4	17, 22, 25, 26, 27
TC	Lenvatinib	31.0	10.0	63.3	20.0	28, 29
	Cabozantinib	15.9	2.4			30
	Vandetanib	9.3	—	36	0	31, 32
HCC	Sorafenib	11.4	1.7	21.8	1.1	33, 34
	Lenvatinib	24.6	5.7	45.7	8.6	33, 34
	Regorafenib	8.6	—			2, 35
	Cabozantinib	—	—	20.6	8.8	36, 37

Abbreviations: A, Asian; HCC, hepatocellular carcinoma; mRCC, metastatic renal cell carcinoma; Ref, reference; TC, thyroid cancer; VEGFR‐TKI, vascular endothelial growth factor receptor‐targeted tyrosine kinase inhibitor.

The reports examined in this review varied in the type of VEGFR‐TKI used, dose, line of treatment, breakdown of prior therapy, and duration of treatment among cancer types, and they did not consider the impact of these differences on the occurrence of proteinuria. The presence or absence of underlying disease and renal function status at the time of VEGFR‐TKI administration should have also been considered. In particular, because nephrectomy is frequently performed as a surgical treatment for mRCC, a significant number of patients with advanced‐stage mRCC treated with VEGFR‐TKIs have a single kidney. One report showed that renal function was reduced after nephrectomy in patients with mRCC,^40^ and another showed that the mean eGFR was significantly higher in patients who underwent partial nephrectomy than in those who underwent radical nephrectomy (56 vs. 44 mL/min/1.73 m^2^), equivalent to grade 3a and 3b chronic kidney disease, respectively.^41^ A history of nephrectomy has been reported as a risk factor for all‐grade proteinuria in patients with mRCC treated with sunitinib and pazopanib.^11^ It is possible that nephrectomy in patients with mRCC does not necessarily affect the appearance of proteinuria, as there are reports suggesting that the appearance of proteinuria is not related to decreased renal function before the start of treatment.^11^, ^42^, ^43^

### Comparison of VEGFR‐TKIs

In all three cancer types studied, there was a trend toward a higher incidence of proteinuria (24%–34%) with lenvatinib than with other VEGFR‐TKIs (Table 1). The aforementioned meta‐analysis showed a significantly increased risk of all‐grade proteinuria with lenvatinib, axitinib, and vandetanib, with an increased risk of high‐grade proteinuria with lenvatinib.^2^ Another network meta‐analysis showed a higher frequency of proteinuria with lenvatinib and axitinib.^44^ A meta‐analysis comparing VEGFR‐TKIs in patients with TC also showed a higher frequency of proteinuria (any grade, grade ≥ 3) with lenvatinib than with sorafenib and vandetanib.^45^ Thus, the high VEGFR1–3 inhibitory activity of lenvatinib may be a factor in the high incidence of proteinuria events with lenvatinib treatment.^46^, ^47^ Regimens combining VEGFR‐TKIs and ICIs for the treatment of mRCC did not show a marked trend toward an increased frequency of proteinuria with the addition of ICIs (Table 2). When the frequency of proteinuria with the combined regimen (ICI + VEGFR‐TKI) was compared with that of sunitinib within the same study, the combined regimen appeared to be associated with a higher frequency of proteinuria. However, the comparison between studies did not necessarily indicate that the combined regimen resulted in a higher frequency of proteinuria than the use of a TKI alone. Therefore, caution should be exercised when interpreting the frequency of proteinuria with the combined regimen.

**TABLE 2:** Incidence of proteinuria in each clinical trial of VEGFR‐TKIs: Comparison between global and Asian patients.

Tumor type	VEGFR‐TKI	G/J	Phase	Total *n*	Control	Prior nephrectomy, %	Proteinuria (Global), %				Proteinuria (Japanese, Asian), %				Ref
							TKI		Control		TKI		Control		
							Any grade	Grade ≥ 3	Any grade	Grade ≥ 3	Any grade	Grade ≥3	Any grade	Grade ≥3	
mRCC	Axitinib	G	3	723	Sor	91	11		7 (Sor)						Rini BI, 2011^12^
	Axitinib	G	3	724	Pbo	100	23		7 (Pbo)						Gross‐Goupil M, 2018^13^
	Axitinib	J	3	54	Sor	96					12	4	10 (Sor)	3.4 (Sor)	Ueda T, 2013^14^
	Axitinib	J/non‐J	2	44 (J)/169	Pbo	84–86	22				64				Oya M, 2017^15^
	Axitinib	J	2	64	—	100					58	9	—	—	Tomita Y, 2011^16^
	Axitinib + pembrolizumab	G	3	861	Sun	83	17.5 (+Pem)	2.8 (+Pem)	11.1 (Sun)	1.4 (Sun)					Rini BI, 2019^17^
	Axitinib + pembrolizumab	J	3	94	Sun	—					40.9 (+Pem)	4.5 (+Pem)	28.6 (Sun)	8.2 (Sun)	Tamada S, 2022^18^
	Axitinib + avelumab	J/G	3	67 (J)/886	Sun	67–80	6.0 (+Ave)	1.6 (+Ave)	3.2 (Sun)	0.9 (Sun)	18.2 (+Ave)	0 (+Ave)	17.6 (Sun)	5.9 (Sun)	Uemura M, 2020^19^
	Cabozantinib	G	3	658	Eve	85	14	2	9 (Eve)	1 (Eve)					Choueiri TK, 2016^20^
	Cabozantinib	J	2	35	—	97					40.0	8.6	—	—	Tomita Y, 2020^21^
	Cabozantinib + nivolumab	G	3	651	Sun	69–71	10.3 (+Nivo)	2.8 (+Nivo)	7.8 (Sun)	2.2 (Sun)					Choueiri TK, 2021^22^
	Lenvatinib + pembrolizumab or everolimus	G	3	1069	Sun	73–77	29.5 (+Pem) 34.1 (+Eve)	7.7 (+Pem) 8.2 (+Eve)	12.6 (Sun)	2.9 (Sun)					Motzer R, 2021^23^
	Lenvatinib + pembrolizumab	J	3	73	Sun	23–26					54.8 (+Pem)	14.3 (+Pem)	25.8 (Sun)	9.7 (Sun)	Eto M, 2023^24^
	Pazopanib	G	3	1110	Sun	82–84	18	4	14 (Sun)	4 (Sun)					Motzer RJ, 2013^25^
	Pazopanib	A	3	367	Sun	81–85					31		29 (Sun)		Guo J, 2018^26^
		non‐A		707		84	8		5 (Sun)						
	Sunitinib	G	3	651	Nivo + Cab	69–71	7.8	2.2	10.3 (Nivo+Cab)	2.8 (Nivo+Cab)					Choueiri TK, 2021^22^
	Sunitinib	G	3	1110	Paz	82–84	14	4	18 (Paz)	4 (Paz)					Motzer RJ, 2013^25^
	Sunitinib	G	3	861	Axi + Pem	83	11.1	1.4	17.5 (Axi + Pem)	2.8 (Axi + Pem)					Rini BI, 2019^17^
	Sunitinib	A	3	367	Paz	81–85					29		31 (Paz)		Guo J, 2018^26^
	Sunitinib	J	3	120	Sor	86					72	4	57 (Sor)	7 (Sor)	Tomita Y, 2020^27^
TC	Lenvatinib	G	3	392	Pbo	—	31.0	10.0	1.5 (Pbo)	0 (Pbo)					Schlumberger M, 2015^28^
	Lenvatinib	J	3	40	Pbo	—					63.3	20.0	0.0 (Pbo)	0.0 (Pbo)	Kiyota N, 2015^29^
	Cabozantinib	G	3	258	Pbo	—	15.9	2.4	2.3 (Pbo)	0 (Pbo)					Brose MS, 2022^30^
	Vandetanib	G	3	205	—	—	9.3	—	—	—					Bastholt L, 2016^31^
	Vandetanib	J	1/2	14	—	—					36	0	—	—	Uchino K, 2017^32^
HCC	Sorafenib	G	3	954	Len	—	11.4	1.7	24.6 (Len)	5.7 (Len)					Kudo M, 2018^33^
	Sorafenib	J	3	168	Len	—					21.8	1.1	45.7 (Len)	8.6 (Len)	Yamashita T, 2020^34^
	Lenvatinib	G	3	954	Sor	—	24.6	5.7	11.4 (Sor)	1.7 (Sor)					Kudo M, 2018^33^
	Lenvatinib	J	3	168	Sor	—					45.7	8.6	21.8 (Sor)	1.1 (Sor)	Yamashita T, 2020^34^
	Regorafenib	G	3	573	Pbo	—	8.6	—	1.0	—					Bruix J, 2017^35^
	Cabozantinib	G	3	707	Pbo	—	—	—	—	—					Abou‐Alfa GK, 2018^36^
	Cabozantinib	J	2	34	—	—					20.6	8.8	—	—	Kudo M, 2021^37^

Abbreviations: A, Asian; Ave, avelumab; Axi, axitinib; Cab, cabozantinib; Eve, everolimus; G, global; HCC, hepatocellular carcinoma; J, Japanese; Len, lenvatinib; mRCC, metastatic renal cell carcinoma; Nivo, nivolumab; Paz, pazopanib; Pbo, placebo; Pem, pembrolizumab; Ref, reference; Sor, sorafenib; Sun, sunitinib; TC, thyroid cancer; VEGFR‐TKI, vascular endothelial growth factor receptor‐targeted tyrosine kinase inhibitor.

### Influence of Japanese/Asian ethnicity

The frequency of proteinuria associated with VEGFR‐TKI administration tends to be higher in Asian populations, including Japanese people, than in other ethnic groups.^11^ In all three cancer types studied, with some exceptions, the frequency of proteinuria in Japanese or Asian populations was markedly higher than in non‐Asian populations, regardless of the type of VEGFR‐TKI or cancer type (Table 1).

Regarding the effect of race by cancer type, a higher frequency of proteinuria was reported in patients with mRCC (Table 2). The phase 3 study comparing sorafenib with sunitinib also reported a high frequency of proteinuria at 57% and 72%, respectively.^27^ In a subgroup analysis of the COMPARZ trial, the frequencies of any‐grade proteinuria in the non‐Asian population were 8% for pazopanib and 5% for sunitinib, whereas in the Asian population, the respective frequencies were 31% and 29%.^26^ In addition, a pooled analysis of two phase 3 trials on mRCC (n = 1392) showed that Asian ethnicity was an independent and significant risk factor for both any‐grade proteinuria and grade ≥3 proteinuria.^11^ For cabozantinib, the frequency of proteinuria in Asian people was also higher than in non‐Asian populations.^20^, ^21^

In the phase 3 CLEAR study, the incidence of proteinuria in patients overall for lenvatinib plus pembrolizumab was 29.5% versus 12.6% for sunitinib, but the incidence of proteinuria was higher in the Japanese subgroup for both treatments (54.8% and 25.8%, respectively).^24^ However, it may be acknowledged that the duration of treatment is more than twice as long for lenvatinib plus pembrolizumab than it is for sunitinib. A Japanese subgroup analysis of the AXIS trial comparing axitinib and sorafenib in patients with mRCC showed no remarkable difference in the incidence of proteinuria between the Japanese subgroup and the overall study population in either the axitinib or sorafenib groups.^14^

In TC, a subgroup analysis of the phase 3 SELECT study of lenvatinib showed a higher incidence of proteinuria in Japanese patients than in the overall population (any grade: 63.3%; grade ≥ 3: 20%) (Table 2).^29^ A recent Korean cohort study also showed a high incidence of proteinuria for lenvatinib, with the incidence being 80% for any‐grade proteinuria and 32% for grade 3/4 proteinuria.^48^ A similar trend toward a higher frequency in Japanese people has been suggested for vandetanib in previous trials.^31^, ^32^

In HCC, the incidence of any‐grade proteinuria in patients treated with lenvatinib and sorafenib was 24.6% and 11.4%, respectively,^33^ while in the Japanese subgroup, the frequency was 45.7% and 21.8%, respectively^34^ (Table 2). It should be considered that the duration of treatment was 5.7 months for lenvatinib and 3.7 months for sorafenib.^33^ Thus, for both TC and HCC, the frequency of proteinuria tended to be higher among Asian and Japanese populations.

In mRCC, a study examining racial differences in the efficacy and safety of VEGFR‐TKIs showed that in addition to proteinuria, adverse events, such as hand‐foot syndrome and hypothyroidism, were more frequent in Japanese people.^19^, ^24^ Therefore, the susceptibility to adverse events with VEGFR‐TKIs may be higher in Japanese people. It is possible that differences in body size, metabolic enzymes, and genetic polymorphisms among races may have affected the results, but the mechanisms for this are not clear.

## RISK FACTORS FOR PROTEINURIA DEVELOPMENT WITH VEGFR‐TKIs (Table 3)^11^, ^16^, ^42^, ^49^, ^50^

**TABLE 3:** Risk factors for proteinuria associated with VEGFR‐TKI therapy.

Risk factor	Study design	*n*	Tumor type	VEGFR‐TKI	Ref
Baseline urine protein concentration	Phase 2	64	mRCC (J)	Axitinib	Tomita Y, 2011^16^
	Retrospective	50	mRCC (J)	Axitinib	Ikesue H, 2022^49^
	Phase 3	1392	mRCC	Sunitinib/pazopanib	Sorich MJ, 2016^11^
Race (Asian)	Phase 3	1392	mRCC	Sunitinib/pazopanib	Sorich MJ, 2016^11^
Diabetes mellitus	Phase 3	1392	mRCC	Sunitinib/pazopanib	Sorich MJ, 2016^11^
Prior nephrectomy	Phase 3	1392	mRCC	Sunitinib/pazopanib	Sorich MJ, 2016^11^
Blood pressure (hypertension)	Phase 3	1392	mRCC	Sunitinib/pazopanib	Sorich MJ, 2016^11^
	Phase 1	168	Solid tumor	TKI	Boissier E, 2017^42^
	Retrospective	124	Solid tumor (J)	TKI	Kanbayashi Y, 2020^50^
Number of cycles (long‐term therapy)	Retrospective	124	Solid tumor (J)	TKI	Kanbayashi Y, 2020^50^
Without RAS inhibitor use	Retrospective	50	mRCC (J)	Axitinib	Ikesue H, 2022^49^
Calcium channel blocker use	Retrospective	124	Solid tumor (J)	TKI	Kanbayashi Y, 2020^50^

Abbreviations: J, Japanese; mRCC, metastatic renal cell carcinoma; RAS, renin–angiotensin system; Ref, reference; VEGFR‐TKI, vascular endothelial growth factor receptor‐targeted tyrosine kinase inhibitor.

Several risk factors associated with VEGFR‐TKI‐induced proteinuria have been reported. Baseline urinary protein concentrations have been demonstrated as a significant risk factor for the occurrence of proteinuria associated with VEGFR‐TKI treatment with axitinib, sunitinib, and pazopanib in patients with mRCC.^11^, ^16^, ^49^ In a pooled analysis of two phase 3 trials involving pazopanib or sunitinib in patients with mRCC, Asian ethnicity, diabetes mellitus, baseline systolic blood pressure, pre‐existing grade 1 proteinuria, and prior nephrectomy were significant independent predictors of proteinuria development.^11^ Asian ethnicity, diabetes mellitus, and hypertension have also been demonstrated as independent risk factors for any‐grade proteinuria, as well as grade 3/4 proteinuria.^11^ It has also been suggested that non‐use of renin–angiotensin system (RAS) inhibitors is a significant risk factor for proteinuria development in patients with mRCC treated with axitinib.^49^

In TC, the duration of administration and the number of cycles of cabozantinib were risk factors for proteinuria development.^51^ Furthermore, older patients (≥65 years) treated with lenvatinib appeared to be more likely to experience grade ≥3 proteinuria than younger patients (13.2% vs. 7.7%, respectively).^52^

In our review of the existing evidence, there was no noticeable trend toward a higher frequency of proteinuria with concomitant use of ICIs, and the evidence for each individual drug was limited; therefore, further studies on the risk factors for proteinuria with each VEGFR‐TKI are needed. Translational studies are expected to be conducted to identify biomarkers for the development of proteinuria induced by VEGFR‐TKIs.

## EFFECTS OF PROTEINURIA ON RENAL FUNCTION (Table 4)

**TABLE 4:** Changes in renal function associated with VEGFR‐TKI therapy.

Study design	*n*	Tumor type	Treatment	Median treatment period (months)	Change in eGFR	Ref
Retrospective	40	TC	Lenvatinib	29.5	Significant (21.0 mL/min/1.73 m^2^) decrease in eGFR at 48 months from baseline	Masaki C, 2021^53^
Retrospective	65	mRCC	Axitinib	12.3–13.3	No significant change in eGFR from baseline, regardless of baseline proteinuria	Miyake H, 2015^54^
Retrospective	73	TC	Lenvatinib, sorafenib	14.9 (lenvatinib) 4.65 (sorafenib)	Mean ΔeGFR was −6.75% with lenvatinib and + 5.90% with sorafenib (not significant)	Iwasaki H, 2019^55^

Abbreviations: eGFR, estimated glomerular filtration rate; mRCC, metastatic renal cell carcinoma; Ref, reference; TC, thyroid cancer; VEGFR‐TKI, vascular endothelial growth factor receptor‐targeted tyrosine kinase inhibitor.

There is limited evidence regarding the relationship between proteinuria development and decreased renal function when using VEGFR‐TKIs. In one study, the effects of VEGFR‐TKIs on renal function were studied in 40 patients with advanced differentiated TC treated with lenvatinib for at least 6 months.^53^ The eGFR showed a sustained decrease, with values of 11.4, 18.3, and 21.0 mL/min/1.73 m^2^ at 24, 36, and 48 months after treatment initiation, respectively. Proteinuria has also been demonstrated as a risk factor for the decrease in eGFR.^53^

When the effect of proteinuria on renal function in patients with mRCC treated with axitinib was investigated, 41 of 65 patients (63.1%) tested positive for proteinuria, but there was no significant difference between the eGFR before axitinib introduction and at the last clinic visit, regardless of whether proteinuria was present.^54^ A retrospective analysis of patients with radioactive iodine‐resistant differentiated TC treated with sorafenib and lenvatinib showed that long‐term VEGFR‐TKI treatment adversely affected renal function. However, the appearance of proteinuria was not a significant factor associated with a reduction in eGFR.^55^

Thus, although no clear trend was observed in the association between proteinuria development and decreased renal function when using VEGFR‐TKIs, the results should be interpreted with caution because of the limited number of reports evaluating the relationship with decreased renal function and the different criteria used to define decreased renal function among the studies. While it is generally established that proteinuria injures the kidneys and reduces renal function,^56^ it is not clear whether proteinuria associated with VEGFR‐TKI administration also reduces renal function. In general, VEGFR‐TKIs are rarely administered for long periods of time. However, using them in combination with ICIs tends to prolong prognosis, as well as the duration of TKI administration. Even when grade 3/4 proteinuria does occur, the effect of proteinuria resulting from VEGFR‐TKIs on renal function may be limited because proteinuria is considered to be basically reversible as it improves with drug withdrawal and dose reduction,^8^ although the exact time taken for proteinuria to improve with drug withdrawal and dose reduction is unclear. The degree to which proteinuria is affected by individual drugs and the extent to which the presence or absence of underlying disease (diabetes mellitus, hypertension) affects renal dysfunction due to proteinuria are not clear and require further investigation.

## MANAGEMENT OF PROTEINURIA ASSOCIATED WITH VEGFR‐TKIS

### Monitoring method

As treatment with VEGFR‐TKIs in various cancer types is becoming more widespread and prolongs the survival of patients with cancer, it is necessary to organize a policy on the management of proteinuria and how to prioritize cancer treatment. First, it is important to note that underlying diseases, such as hypertension and diabetes mellitus, which are common risk factors for proteinuria, must be adequately controlled before the initiation of anticancer drug treatment.^11^ Second, appropriate proteinuria monitoring when using VEGFR‐TKI treatment is important. Proteinuria occurs soon after treatment initiation in most cases; therefore, it is important to start monitoring after treatment initiation^57^ and to check for changes from baseline early (after about 2 weeks).

Monitoring is performed by qualitative (urine protein dipstick testing) and quantitative (urinary protein creatinine ratio [UPCR], 24‐hour urine storage) methods. In a report comparing the evaluation results of the urine protein dipstick method and the UPCR, even when the evaluation result of the urine protein dipstick method (dipstick testing) was 2+ or 3+, the UPCR showed a lower value in some cases.^58^, ^59^ Therefore, the evaluation should be based on both quantitative and qualitative tests.

### What to do at the onset of proteinuria

If a trend toward proteinuria is observed, the first step is to take general measures, such as avoiding dehydration, discontinuing the use of potentially nephrotoxic medications, and controlling blood pressure. To avoid dehydration, lifestyle modifications should be implemented, such as limiting salt intake and increasing fluid intake.

If grade 3/4 proteinuria develops, the patient should be treated in accordance with the withdrawal/dose reduction criteria for the appearance of proteinuria for each VEGFR‐TKI, and after recovery of proteinuria, the patient should consider whether to restart the drug. In the case of lenvatinib, sunitinib, pazopanib, and sorafenib, grade 4 proteinuria warrants discontinuation.

In a retrospective single‐center analysis conducted in Japan, in patients with refractory differentiated TC with normal renal function prior to VEGFR‐TKI treatment, all but two diabetic patients resumed or continued treatment after dose reduction or withdrawal for patients with reduced renal function following lenvatinib treatment.^55^ In addition, in a retrospective analysis of sunitinib for mRCC, proteinuria improved with withdrawal or dose reduction after the onset of proteinuria.^43^ These results suggest that proteinuria with VEGFR‐TKIs is reversible.

If patients have mildly impaired renal function before starting treatment with VEGFR‐TKIs, it is considered unnecessary to adjust the starting dose of VEGFR‐TKIs. However, if renal function is moderately to severely impaired, dose adjustment may be considered when starting treatment. However, the relationship between the starting dose of VEGFR‐TKIs and proteinuria has not been fully verified, and more evidence needs to be generated in the future.

In addition, because VEGFR‐TKIs are often used in patients with advanced disease; that is, stage IV, there may be cases where treatment continuation should be a priority, such as in symptomatic cases. The decision to continue or discontinue treatment is based on whether the benefits of continuation outweigh the risks associated with proteinuria progression.

The current primary treatment for mRCC is the combination of VEGFR‐TKIs and ICIs. There are reports showing that prolonged TKI administration and PD‐1 inhibitors are predictors of proteinuria development, and that prolonged TKI administration is a prognostic predictor of decreased renal function.^60^ Moreover, the combination of an ICI and VEGFR inhibitor compared with sunitinib alone increased the risk of proteinuria development.^61^, ^62^ Therefore, the occurrence of proteinuria may be due to a direct effect of concomitant use of an ICI with a VEGFR inhibitor, or an indirect effect of a prolonged duration of TKI administration. With combined therapy (ICI + VEGFR inhibitor), the ICI can be continued during the VEGFR‐TKI withdrawal period. Therefore, the VEGFR‐TKI can be withdrawn for a long period of time and the patient's progress followed up until proteinuria has sufficiently recovered. In light of the above, more careful monitoring for proteinuria is needed than was necessary before the advent of ICIs.

Referral to a nephrologist should be considered for patients with symptoms related to decreased renal function, such as edema, or when proteinuria has not improved after medication withdrawal. A nephrologist should also be consulted when renal function, as assessed by eGFR, is impaired or when thrombotic microangiopathy is suspected.

### Control of risk factors for proteinuria

Risk factors for proteinuria should also be controlled. Hypertension is a risk factor for proteinuria development during VEGFR‐TKI use; therefore, its control is important.^8^ If hypertension is present before treatment or after treatment initiation, RAS inhibitors, such as angiotensin‐converting enzyme inhibitors and angiotensin II receptor blockers, are the first choice.^8^

There are limited studies on whether the renoprotective effects of RAS inhibitors are also exerted in the presence of VEGFR‐TKIs. A study in rats showed that axitinib plus candesartan, an angiotensin receptor blocker, improves the UPCR more than axitinib alone.^63^ In addition, in a retrospective study of mRCC patients treated with axitinib in real‐world clinical practice, of 28 patients treated with antihypertensive medications, 17 patients taking RAS inhibitors had a significantly lower incidence of grade ≥2 proteinuria (*p* = 0.001).^49^ At present, there is no evidence suggesting that RAS inhibitors are effective in normotensive patients.^8^ Baseline diabetes mellitus is also a risk factor for proteinuria, but the treatment of diabetes mellitus should be independent of cancer treatment with VEGFR‐TKIs and is excluded from the discussion in this review.

## CONCLUSION

Although treatment with VEGFR‐TKIs contributes to the long‐term survival of patients with cancer, the increased risk of proteinuria in patients treated with VEGFR‐TKIs requires appropriate management. In this narrative review, the incidence of proteinuria was examined based on phase 3 trials of VEGFR‐TKIs in mRCC, TC, and HCC. The results revealed that the frequency of proteinuria did not differ significantly by cancer type, and that the incidence of proteinuria was significantly higher in certain ethnic groups, such as Asians and in particular, Japanese. Although there was a trend toward a higher incidence of proteinuria with lenvatinib than with other VEGFR‐TKIs, the interpretation should consider the difference in the duration of treatment. In terms of proteinuria management, when grade 3/4 proteinuria occurs, the patient should be treated according to the criteria for dose reduction or withdrawal specified for each drug. For grade 2 proteinuria, treatment continuation is chosen when the benefits of continued treatment outweigh the risks. In addition, regular monitoring of proteinuria is essential to achieve appropriate management. These systematic management practices should be implemented universally, regardless of the type of cancer.

## AUTHOR CONTRIBUTIONS

**Taigo Kato**: Writing—original draft; Methodology; Investigation. **Ryuichi Mizuno**: Writing—review & editing; Data curation. **Hideaki Miyake**: Writing—review & editing; Conceptualization; Supervision.

## CONFLICT OF INTEREST STATEMENT

TK received grants from Takeda Pharmaceutical Co., Ltd., and Pfizer Japan Inc.; honoraria from Takeda Pharmaceutical Co., Ltd., Pfizer Japan Inc., Merck Ltd., MSD K.K., and Astellas Pharma Inc.; and payment for expert testimony from Eisai Co., Ltd. RM received honoraria from Bristol‐Myers Squibb K.K., Eisai Co., Ltd., Merck Ltd., MSD K.K., Ono Pharmaceutical Co., Ltd., and Pfizer Japan Inc. HM received honoraria from MSD K.K., Takeda Pharmaceutical Co., Ltd., and Eisai Co., Ltd.

## INFORMED CONSENT

N/A.

## ANIMAL STUDIES

N/A.

## FUNDING INFORMATION

The authors received payment for participation in an advisory board meeting, but the present manuscript was prepared independently with funding for medical writing only. Taigo Kato reports research funding from Takeda Pharmaceutical Co., Ltd., and Pfizer Japan Inc., outside the submitted work.

## INSTITUTIONAL REVIEW BOARD APPROVAL FOR THE RESEARCH PROTOCOL

N/A.

## REGISTRY AND THE REGISTRATION NO. OF THE STUDY/TRIAL

N/A.
